# The Science of Soy: What Do We Really Know?

**DOI:** 10.1289/ehp.114-a352

**Published:** 2006-06

**Authors:** Julia R. Barrett

A stroll through nearly any American grocery store or pharmacy yields ample proof of the soybean’s increasing role in the U.S. diet. Food packaging offers statements about products’ soy content and the purported associated health benefits. Products such as tofu, soy milk, soy-based infant formula, and meatless “texturized vegetable protein” burgers are widely available. Shelves of dietary supplements and nutraceuticals are stocked with isoflavones, naturally occurring estrogenic compounds found in soy. The general impression is one of certainty that both soy and soy isoflavones deliver many health benefits, including prevention of cardiovascular disease, cancer, and osteoporosis, as well as treatment of menopausal symptoms. The science is less absolute, however, and still evolving.

Soy provides a complete source of dietary protein, meaning that, unlike most plant proteins, it contains all the essential amino acids. According to the American Soybean Association, 3.14 billion bushels (85.5 million metric tons) of soybeans were harvested in the United States in 2004. Approximately half of the harvest was exported, and most of the remainder was crushed to produce oil and protein meal for domestic use. An April 2006 report from the USDA Economic Research Service indicates that only a small amount of whole soybeans are used to produce soy foods, and just 2% of soy protein meal is used for human consumption; the rest is used for animal feed.

The Soyfoods Association of North America reports that U.S. sales of soy foods reached $3.9 billion in 2003, continuing an 11-year trend of 15% average annual increases. According to the United Soybean Board’s 2004–2005 *Consumer Attitudes About Nutrition* report, 25% of Americans consume soy foods or beverages at least once per week, and 74% view soy products as healthy.

Nevertheless, Americans as a whole still consume very little soy protein. Based on 2003 data from the UN Food and Agriculture Organization, per-capita soy protein consumption is less than 1 gram (g) per day in most European and North American countries, although certain subpopulations such as vegetarians, Asian immigrants, and infants fed soy-based formula consume more. The Japanese, on the other hand, consume an average 8.7 g of soy protein per day; Koreans, 6.2–9.6 g; Indonesians, 7.4 g; and the Chinese, 3.4 g.

Traditional soy foods include tofu, which is produced by puréeing cooked soybeans and precipitating the solids, and miso and tempeh, which are made by fermenting soybeans with grains. “Second generation” soy products involve chemical extractions and other processing, and include soy protein isolate and soy flour. These products become primary ingredients in items such as meatless burgers, dietary protein supplements, and infant formula, and are also used as nonnutritive additives to improve the characteristics of processed foods.

## Health Effects of Soy

Soybeans and soy foods contain a variety of bioactive components, including saponins, protease inhibitors, phytic acid, and isoflavones. Isoflavones belong to a class of compounds generally known as phytoestrogens, plant compounds that have estrogen-like structures.

The dominant isoflavone in soy is genistein, with daidzein and glycitein composing the remainder. Within soy, isoflavones are almost entirely bound to sugars, producing the respective compounds genistin, daidzin, and glycitin. Soy isoflavones have been linked with numerous health effects, but the strength of the relationships and whether the effects are beneficial are strongly debated.

Soy isoflavones are frequently referred to as weak estrogens, and depending upon the specific circumstance, they can act as agonists, partial agonists, or antagonists to endogenous estrogens (such as estradiol) and xenoestrogens (including phytoestrogens) at estrogen receptors. They are not especially potent, however, and activity varies by tissue concentration, cell type, hormone receptor type, and stage of differentiation. In addition to their estrogen receptor activity, isoflavones may also interfere with steroid metabolism by inhibiting aromatase, hydroxysteroid dehydrogenase, and steroid α-reductase, and by altering the ratio of estradiol metabolites. Soy isoflavones may also act as antioxidants; inhibitors of proteases, tyrosine kinases, and topoisomerases; inducers of Phase I and/or Phase II enzymes such as cytochrome P450s, glutathione *S*-transferase, and quinone reductase; and inhibitors of angiogenesis.

Such activities have potential benefits—if they occur in the body. Caution is necessary when predicting *in vivo* potency from *in vitro* systems. *In vitro* systems are valuable for investigating the structure–activity relationships and the mechanisms of isoflavone actions, but *in vitro* tests have used genistein concentrations that may be five times higher than the peak concentrations seen in human serum, 95% of which occurs as glucuronide conjugate. Animal studies also require careful extrapolations due to how exposure occurs, interspecies differences in metabolism, and comparability of the stage of development at which exposure occurs.

Retha R. Newbold, a supervisory research biologist at the NIEHS, is well aware of these factors. Concerns about genistein’s effects on reproduction and development are due in part to her extensive research in mice. Newbold believes caution is warranted, because her studies, as well as others, have shown that genistein has such effects as inducing uterine adenocarcinoma in mice and premature puberty in rats. A recent study led by biologist Wendy Jefferson in Newbold’s laboratory and published in the October 2005 issue of *Biology of Reproduction* linked genistein with effects such as abnormal estrous cycle, altered ovarian function, and infertility in mice.

The original interest in soy was fueled by geographic epidemiology—the observation that populations that consume a lot of soy, particularly those in eastern Asia, have less breast cancer, prostate cancer, and cardiovascular disease, and fewer bone fractures. Additionally, women in these populations report fewer menopausal symptoms, such as hot flashes, and both men and women have a lower incidence of aging-related brain diseases. Since lifestyle can affect chronic disease development, and diet is a major lifestyle factor, traditional Asian diets drew considerable attention.

Although initial research overestimated the amount of soy consumed by Asians, the cumulative evidence of numerous biomarker studies has confirmed that their diets are significantly higher in both isoflavones and lignans (another phytoestrogen) compared to the typical Western diet. Studies have further shown that when Asians emigrate to Western nations such as the United States and adopt the prevailing diet, their disease rates change.

What’s interesting, says Jay Kaplan, head of comparative medicine at Wake Forest University School of Medicine, is that people who switch to an American-style diet from a traditional diet high in plant protein take on the disease characteristics of the host population, not those of their ancestral population. “It does seem to be something that’s in the environment, and it looks like this reliance on plant proteins is one of these things that goes away after [immigrants have] been here a while,” Kaplan says. “What also goes away is any protection from chronic disease that we ascribe to those populations.”

By the late 1990s, the epidemiologic and experimental data seemed strong enough to support recommendations to incorporate soy in the diet. Still, not everyone was convinced.

## Isoflavone Variables and Risks

Soy research is complicated because there’s considerable variation in isoflavone exposure among people classified as soy consumers. Agronomic factors (such as the soybean cultivar and the environmental conditions under which the crop grew) affect a food’s isoflavone profile, as does the way a soy food is processed. For example, soy protein concentrate produced by alcohol extraction may have only 12.5 milligrams (mg) total isoflavones per 100 g, in contrast to the nearly 199.0 mg total isoflavones per 100 g of full-fat roasted soy flour. Additionally, the fact that most of the isoflavones in food occur bound to sugar affects how they are digested.

Once genistin enters the digestive tract, it releases its sugar and becomes “free” genistein. Some of this free genistein is absorbed. However, most is reconjugated into glucuronides or sulfates, the primary circulating forms of genistein, which are thought to have either low or no biological activity. Only a very small amount of free genistein escapes conjugation by the liver and circulates in that form.

“People need to know that as it occurs in soy and other plant products, genistin is the compound that’s there. The amount of actual genistein is very low, one percent or less probably,” says Michael Shelby, director of the National Toxicology Program’s Center for the Evaluation of Risks to Human Reproduction (CERHR). Key exceptions are fermented products, such as miso and tempeh, which may contain up to 40% free genistein.

Several researchers say that figuring out the pharmacokinetics of genistin and genistein is a vital piece of missing information. “It’s a matter of finding out how much genistin is converted to genistein in the digestive system, and that information is not known,” says Jefferson. “I don’t think a lot of this was understood years ago when some of the animal experiments started, and at that time we didn’t have a clear understanding of the metabolism and fate of these chemicals. We did the best we could, as a community, to try to use the compound we thought would be the one we should look at. I think it’s given us some excellent starting types of data, where we know that these compounds are capable of causing reproductive and developmental effects.”

According to Thomas Badger, director and senior investigator at the Arkansas Children’s Nutrition Center in Little Rock, however, these effects are seen only under certain experimental conditions that are not likely to occur in humans—and therein lies the crux of the debate. Criticisms of many studies of genistein’s effects on reproduction and development have centered on exposure occurring by injection and consequently bypassing the usual metabolic pathways. There is also disagreement about the use of neonatal mice—commonly used in studies of reproduction and development—as a suitable model for predicting effects in human infants.

Despite these criticisms, Newbold stands by her data. “There was some confusion on the fact that in all of our work we have injected genistein,” she says. “We went back and did some of the pharmacokinetics with that to show that the total circulating amounts of genistein are very similar to what’s been reported in feeding rats and also in infants. Metabolism doesn’t have to be the same, but you have to know that the active compounds are getting to the target tissue. Ultimately, a mouse and a rat are not the human, though. You just have to accept it and be as careful with your extrapolations as possible.”

Further controversy surrounds the fact that most of the epidemiologic studies of Asian populations involved whole soy foods, but animal and human intervention studies have generally used soy concentrates or isolated isoflavones; some animal studies used pure genistein. This difference may have obscured what the health effects of soy actually are.

“I’m reasonably sure that any time you take one of those isoflavones and give it separately, you don’t see the same effects as when all three of the isoflavones of soy are given,” says Kaplan. “Based on everything that we know, the best health effects probably come from the whole isolated soy protein given together. There’s something about the intact product that seems to be bioactive that is not able to be replicated when you begin chopping it up.”

In *Expert Panel Report on the Reproductive and Developmental Toxicity of Genistein,* a March 2006 review of the literature on this compound, an expert panel convened by the CERHR scrutinized what has been learned about human exposure to genistein and the associated reproductive and developmental consequences. The most highly exposed adult population was Japanese, with a daily average intake of 0.43 mg per kilogram body weight, which was approximately 10-fold less than the no-effect levels found in rodent studies. Based on the conclusions presented at a meeting held on 15–17 March 2006, the panel found little cause for concern about human exposure to genistein. However, no consideration was made for the amount of genistin found in the diet or how much of it is hydrolyzed in the digestive system to genistein. Further, the panel’s conclusions were not unanimous.

Considerably less attention has attached to daidzein, though there are currently indicators that it may play a larger role than genistein in soy’s apparent beneficial health effects. Like genistein, daidzein in soy exists primarily in linkage with a sugar molecule. This complex, daidzin, is hydrolyzed and the sugar molecule removed in the gut. Daidzein can also be conjugated to glucuronic acid or sulfate in the gut and liver. It may also be converted to equol (suspected of having a higher estrogenic potency than the original daidzein) by gastrointestinal bacteria. There is considerable variability in individuals’ ability to produce equol, and the metabolic pathways for both genistein and daidzein may vary due to factors such as a person’s particular microflora, intestinal transit time, and current or recent use of antibiotics and other drugs.

Thomas Clarkson, a professor of comparative medicine at the Wake Forest University School of Medicine, points out that although soy protein has a very large beneficial effect on cardiovascular health in monkeys, the effects are much less clear in women. Daidzein metabolism may be the key.

“Our best clue is that all monkeys are equol producers, but only about twenty-five or thirty percent of women are equol producers,” Clarkson explains. “There’s some suggestion now that those women who are equol producers do derive some cardiovascular benefits. The fact that [the effects are] so profound in monkeys may have to do with the fact that they’re all equol producers, and [those effects] may only be translatable to the women who are equol producers.”

There have been only a few studies that have looked exclusively at glycitein, the third soy isoflavone, but those have not been on health effects. There are indicators from a couple of recent *in vitro* studies that glycitein may be protective of bone. Most glycitein research has focused on determining how to detect the compound, and its estrogenicity and metabolism.

## Soy-Based Infant Formula

Approximately 20–25% of U.S. infants receive at least some soy-based formula in their first year (there are no numbers on how many are exclusively fed soy formula). Unlike soy milk, which is sometimes mistakenly—and tragically—used in its place, soy formula contains soy protein isolate supplemented with additional amino acids, minerals, vitamins, and fats necessary to support infant growth and development. Parents may choose soy formula for babies who are allergic to cow’s milk–based formula or if they themselves do not consume dairy products.

Steroid hormones affect myriad processes during development, including the formation of hormone-responsive tissues and organizing and activating effects in the central nervous system. Some researchers are therefore concerned that isoflavones from soy-based infant formula might perturb that system, with long-term consequences.

In a study led by Kenneth Setchell at the Children’s Hospital Medical Center in Cincinnati and published 5 July 1997 in *The Lancet*, infants fed soy formula were found to receive 28–47 mg of soy isoflavones per day. Isoflavones were detected in blood, showing that infants absorbed the compounds from the intestine. This was not a given, since the infant gut is significantly different from the adult gut and continues to develop through the first year.

Biological effects are plausible but not necessarily detrimental. For example, in a study comparing the short-term, long-term, and multigenerational effects of soy protein isolate, casein, and whey on the health and development of rats, a group led by Badger found that soy protein isolate was protective against chemically induced breast cancer. This study appeared in the 1 May 2001 issue of the *International Journal of Toxicology*.

In advising caution in feeding infants soy formula, several groups cite a study led by Richard Sharpe at the Centre for Reproductive Biology in Edinburgh, Scotland. The study, published in *Human Reproduction* in July 2002, compared infant marmosets fed cow’s milk–based formula with others that were fed soy-based formula. The soy-fed marmosets had comparatively lower testosterone levels and higher numbers of Leydig cells per testis. However, a follow-up study published in April 2006, also in *Human Reproduction*, indicated no obvious effects on reproduction.

One of the few human studies was led by Brian Strom of the University of Pennsylvania in Philadelphia, in conjunction with the University of Iowa, and published in the 15 August 2001 issue of *JAMA*. In this study, adults who had been in a controlled feeding study during infancy completed a telephone interview about their health, development, and reproductive history. The only significant differences reported were that women who received soy formula as infants had slightly longer menstrual bleeding and more discomfort than women who had received cow’s milk–based formula. A follow-up letter to the *JAMA* editor pointed out that both the Strom study and a retrospective epidemiological study published in the April 1990 issue of the *Journal of the American College of Nutrition* suggested that consumption of soy formula could adversely affect immune function in children.

More human data are clearly needed, as described in the recent CERHR *Expert Panel Report on the Reproductive and Developmental Toxicity of Soy Formula*. “The findings for soy formula were that there’s just not enough information to make the call. I’m not surprised by that at all,” says Newbold. “Hopefully, the next step that will come from this is that there certainly will be more research with soy formula and more epidemiology studies. That’s definitely what we’re missing.”

For their part, both Jefferson and Newbold caution against using their results to determine the safety of soy formula, although they believe their findings of adverse effects in rodents provide strong evidence that concern is warranted. Their findings are not definitive proof that soy formula is harmful, however. “The studies in our laboratory are to determine if these compounds can cause an effect at any dose level,” says Jefferson. “The studies we do in our laboratory were designed to study mechanism, and not specifically intended for risk assessment.”

Given the limited evidence for the health effects of soy isoflavones in infants, pediatric and health organizations in several countries suggest caution in feeding soy to infants and young children. If an infant is not receiving breast milk (either its mother’s or a donor’s), cow’s milk–based infant formula is the first recommendation. If there seems to be a problem with that option, parents shouldn’t automatically switch to soy formula, assuming dairy allergy or lactose intolerance. Soybeans are a major allergen, and a significant percentage of children who are sensitive to dairy are also sensitive to soy.

However, other experts indicate that soy formula is an adequate source of nutrition for infants with more than 40 years of apparently safe use. “It’s always true that there could be something subtle that we didn’t look for or didn’t know to look for, but so far we haven’t seen any major health problems,” says Susan Baker, a pediatric gastroenterologist at the Children’s Hospital of Buffalo and former chair of the American Academy of Pediatrics Committee on Nutrition. Marisa Salcines, director of communications for the Atlanta-based International Formula Council, which represents infant formula manufacturers, adds that there’s no conclusive evidence for alarm in terms of genistein in soy formula. “There haven’t been any studies that have shown any negative effects in adults who consumed soy-based infant formulas as babies,” she says.

To clarify whether or not concerns are justified, Badger is leading the world’s largest longitudinal, prospective study of children comparing soy-based formula, cow’s milk–based formula, and breast milk. During the study, now in its fourth year, children receive multiple in-depth checkups, including assessments of bone development and health, imaging of reproductive tissues, and assessments of brain development and function, metabolism, growth, development, and body composition. The research team aims to enroll 600 pregnant women, whose children will be followed from birth through puberty.

Additionally, Walter Rogan, a senior investigator in the NIEHS Epidemiology Branch, is heading the Study of Estrogen Activity and Development. Through pilot studies conducted in late 2004 at the Children’s Hospital of Boston and the Children’s Hospital of Philadelphia, researchers gathered physical, sonographic, and biochemical data from infants fed soy formula, cow’s milk formula, or breast milk. Data analysis is currently under way.

## Finessing Investigations

On balance it does not seem that soy and its constituent isoflavones have met original expectations. Clinical results with regard to soy’s ability to reduce the risk of cardiovascular disease have been inconsistent; a review in the 21 February 2006 issue of *Circulation* indicated there was little to no effect. The only apparent impact of soy and soy isoflavones on cardiovascular disease risks seems to be a slight reduction in low-density lipoproteins in individuals who had very high levels of cholesterol. An August 2005 report from the DHHS Agency for Healthcare Research and Quality, *Effects of Soy on Health Outcomes*, also concluded that there was little evidence to support a beneficial role of soy and soy isoflavones in bone health, cancer, reproductive health, neurocognitive function, and other health parameters.

Nevertheless, there remain tantalizing clues that soy may benefit human health. For example, *in vitro* studies with human breast cancer cells suggest that genistein may induce detoxification enzymes and inhibit growth of both estrogen receptor–positive and estrogen receptor–negative cancers. Additionally, *in vitro* studies demonstrate that genistein inhibits prostate cancer cell growth, and epidemiologic studies continue to find an inverse relationship between consumption of isoflavone-rich foods and prostate cancer. Rodent models and *in vitro* systems have suggested beneficial effects on bone density; similar results have not been observed in humans, although clinical trials have shown a promising effect on biomarkers of bone turnover.

Although there has been comparatively little research on the effects of soy and isoflavones on cognition and other brain activity, Clarkson says this area may also hold some promise. “Our group has done some work [in monkeys] showing that [soy] modifies serotonin metabolism in a direction that should be useful in the prevention of depression,” he says.

Michael R. Adams, a professor of pathology at Wake Forest University School of Medicine, has expanded the scope of the research beyond isoflavones. He is currently looking at one of soy’s protein fractions, 7S, which may have a role in inhibiting the development of atherosclerosis by acting directly on the artery wall rather than on plasma lipids or low-density lipoprotein cholesterol receptors.

What most researchers do agree on is that we are only just beginning to truly understand the nature of soy, and that much more research is needed before it is possible to make firm health recommendations. “If you look at nutritional research in general,” Kaplan says, “there are kinds of proteins that are described as being ‘bioactive.’ Most people had assumed that if soy is bioactive, it’s because of the isoflavones. We’re no longer certain of that at all.”

## Figures and Tables

**Figure:**
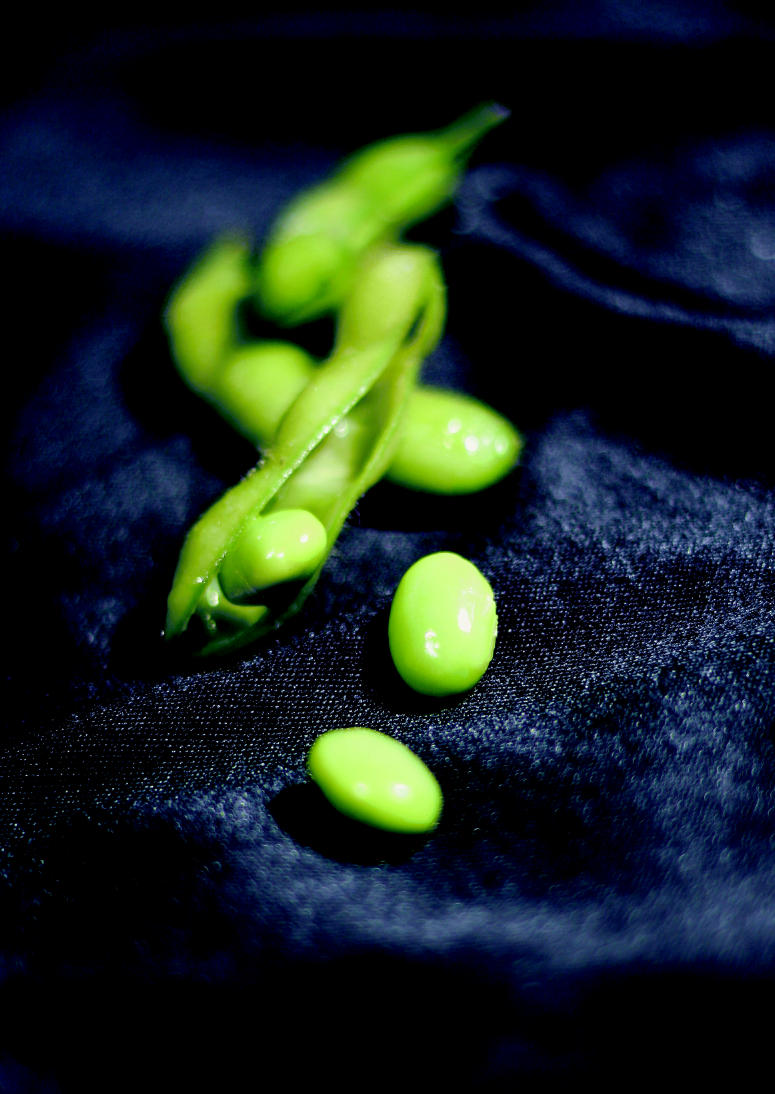


**Figure:**
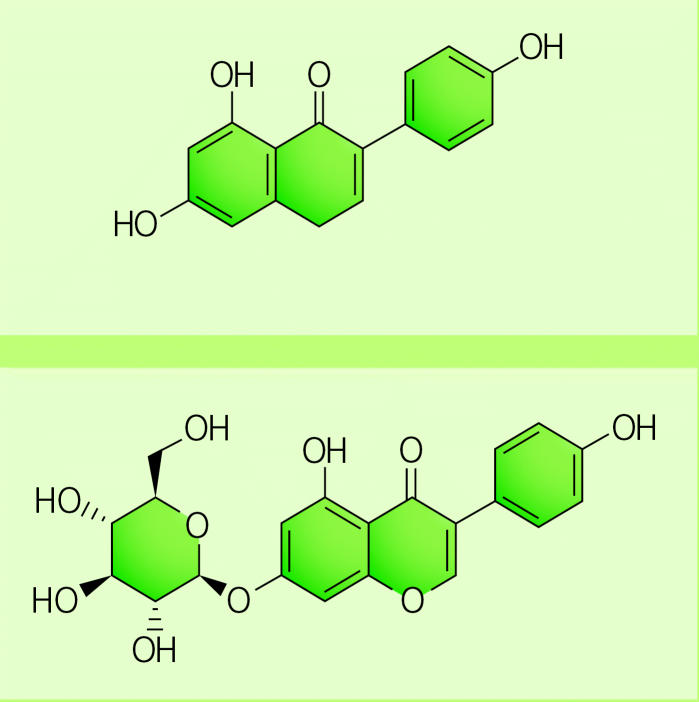
G-force The dominant isoflavone in soy is genistein (above), which within soy is almost always bound to a sugar molecule, producing genistin (below). Once genistin enters the digestive tract, it releases its sugar. Most of the “free” genistein is subsequently reconjugated into glucuronides or sulfates.

**Figure:**
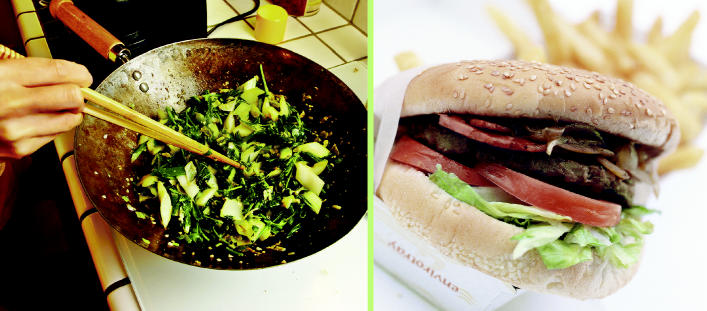
The culture question Early research looked to the differences in soy consumption between Asian and Western diets to explain differences in disease rates, but results are far from conclusive.

**Figure:**
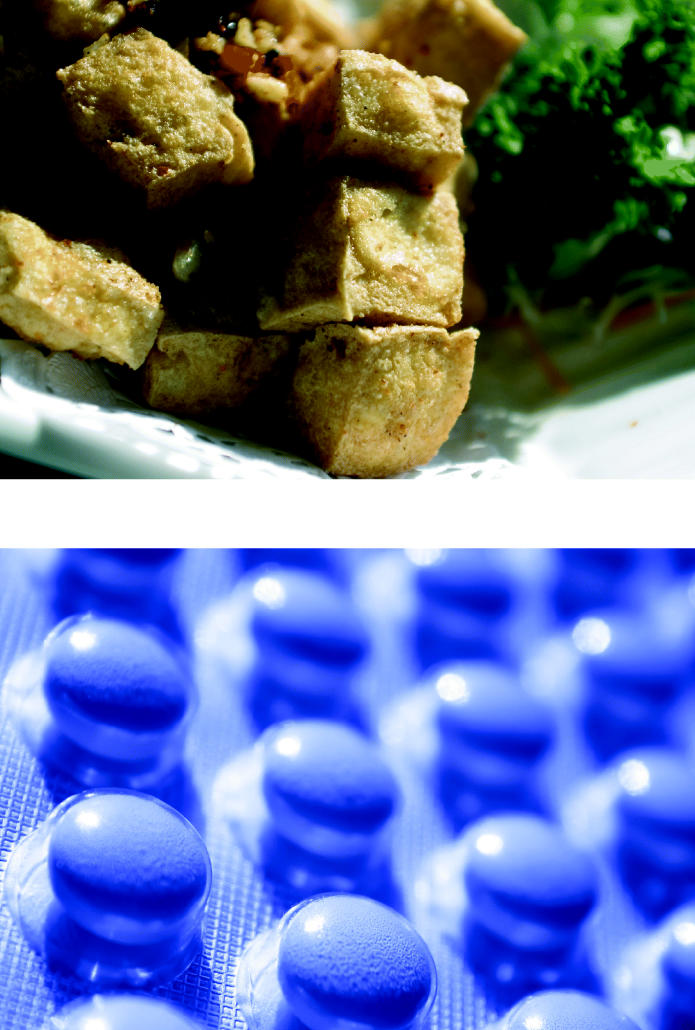
Boon or bane? The touted benefits of consuming soy as part of a healthy diet or ingesting soy supplements as a remedy for menopause symptoms are many, but some data suggests cause for concern.

**Figure:**
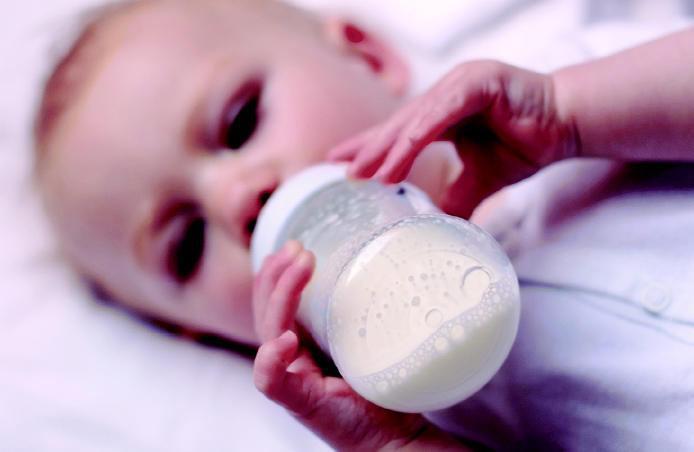
Bottled ‘beans About one-quarter of U.S. children receive some soy-based formula. Parents sometimes choose soy formula in the belief that it is less allergenic than cow’s milk–based formula, even though soybeans themselves are a major allergen.

**Figure:**
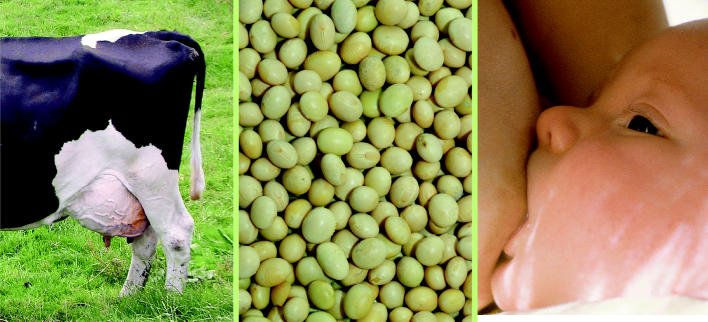
Baby steps toward better understanding A prospective longitudinal study now in its fourth year seeks to clarify whether concerns about soy-based infant formula are justified. The study compares the growth and development of children fed cow’s milk–based formula, soy-based formula, or breast milk, from birth through puberty.

